# Correlation of Mast Cell and Angiogenesis in Oral Lichen Planus, Dysplasia (Leukoplakia), and Oral Squamous Cell Carcinoma

**DOI:** 10.5041/RMMJ.10438

**Published:** 2021-04-29

**Authors:** Amutha Sundararajan, Rajmohan Muthusamy, Kumar Gopal Siva, Prasad Harikrishnan, Sri Chinthu Kenniyan Kumar, Selva Kumar Rathinasamy

**Affiliations:** 1 Oral Pathology and Microbiology, Thai Moogambigai Dental College and Hospital, Mugappair, Chennai, Tamil Nadu, India; 2 Oral Pathology and Microbiology, K.S.R. Institute of Dental Science and Research, Kuchipalayam Post, Tiruchengode, India; 3 Oral and Maxillofacial Surgery, Asan Memorial Dental College and Hospital, Keerapakkam Village, Asan Nagar, Chengalpattu, India

**Keywords:** Angiogenesis, anti-CD34, lichen planus, mast cells, oral squamous cell carcinoma, various grades of dysplasia

## Abstract

**Objective:**

The aim of this study was to compare and correlate mast cell density (MCD) and microvessel density (MVD) between normal oral mucosa, oral lichen planus, various grades of dysplasia, and oral squamous cell carcinoma (OSCC).

**Materials and Methods:**

The study comprised a total of 75 samples, of which 65 were archival tissue blocks of histopathologically confirmed cases, which included 10 cases of oral lichen planus, 25 cases of dysplasia (mild [*n*=10], moderate [*n*=10], and severe [*n*=5]), and 30 cases of OSCC (well differentiated [*n*=10], moderately differentiated [*n*=10], and poorly differentiated [*n*=10]), and 10 samples of normal oral mucosa. All the sections were immunohistochemically stained with anti-CD34 and counterstained with toluidine blue stain. Mean MCD and MVD were determined and analyzed using ANOVA test and compared between the lesions using Tukey HSD test. Pearson’s correlation coefficient test was used to correlate these two factors between various lesions.

**Results:**

Mean MCD and mean MVD were found to be increased in all the lesions compared to normal oral mucosa, and the values were statically significant. Overall, MCD and MVD showed a significant positive correlation (*r*=0.640).

**Conclusion:**

Increase of MCD and MVD and their positive correlation in all the lesions have emphasized their role in the pathogenesis and disease progression.

## INTRODUCTION

Oral squamous cell carcinoma (OSCC) is one of the most common carcinomas of oral cavity in India.^1^,^2^ It develops from the combined influences of an individual’s genetic predisposition and exposure to environmental carcinogens, and is thought to be a multistep process.^2^ Most of the OSCC develop from oral potentially malignant disorders (OPMDs). The rate of malignant transformation of leukoplakia to squamous cell carcinoma ranges from 0.13% to 2.2% per year.^3^ There still remains a controversy in the literature whether patients with oral lichen planus carry an increased risk of developing squamous cell carcinomas. So, better understanding of pathogenesis and disease progression will help to improve targeted therapies against mast cells and angiogenesis in the treatment of these lesions, thereby improving the survival rate of these patients.

Tumor growth/progression is not only determined by the malignant tumor cells; various other cell types and the extracellular matrix (ECM) of the tumor tissue are also involved. It has been proven that tumor growth and metastasis are angiogenesis-dependent. So, continuous recruitment of new capillary blood vessels will be needed for tumor growth.^4^

Angiogenesis is controlled by pro-angiogenic and anti-angiogenic factors. The induction of a tumor vasculature is termed as an “angiogenic switch.” When there is an imbalance between pro-angiogenic and anti-angiogenic factors, the “angiogenic switch” will turn “on” and favors angiogenesis.^5^ Induction of the angiogenic switch depends on the tumor type and the environment.^6^ The tumor cells recruit blood vessels by producing diffusible angiogenic factors that directly activate endothelial cells, thereby stimulating them to sprout and grow towards the developing tumor. They also elaborate cytokines, which attract and activate macrophages, mast cells, and neutrophils, which in turn enhance the angiogenic factors, and they also block the production of angiogenic inhibitors.^7^ Thus, these inflammatory cells also induce neoangiogenesis, promoting tumor progression and metastasis. Tumor-associated macrophages and mast cells play an important role in the progression of many aggressive tumors.^8^

Mast cells play various roles in extracellular matrix degradation, angiogenesis, and innate and acquired immune responses by releasing specific products such as chymase, basic fibroblast growth factor (bFGF), tryptase, heparin, histamine, and cytokines.^9^ Many studies have proved that mast cells accumulate in many angiogenesis-dependent situations, both in physiological and pathological situations like rheumatoid arthritis, wound healing, ovulation, and hemangioma,^10^ and around the tumor margins, as well as releasing pro-angiogenic and angiogenic factors which play a major role in tumor progression.^11^,^12^

Though there are many studies associating mast cells and angiogenesis in many oral lesions (squamous cell carcinoma/dysplasia/lichen planus separately, or combining any of these lesions) (Table 1), to the best of our knowledge none of the studies has correlated all these lesions together. No studies have correlated these two factors in lichen planus so far. So, in our present study, we have made an attempt to associate and correlate the two factors (mast cells and angiogenesis) in normal oral mucosa, oral lichen planus, various grades of dysplasia, and OSCC for better understanding of their role and association in pathogenesis and disease progression.

**Table 1:** Summary of Published Series Reporting the Role of Mast Cells and Microvessels in Various Oral Lesions.

Year	Lesions Included in Study	Factors Compared	Study Findings
2007^13^	Dysplasia Submucous fibrosis OLP OSCC	MC count	MC increased in all the four conditions compared to normal oral mucosa
2008^14^	Dysplasia OSCC	MCD MVD	MVD and MCD seem to be significantly ↑ from normal oral tissues through leukoplakia with various degrees of dysplasia to OSCC
2010^15^	OLP	MCD	MCD in lichen planus ↑ when compared to NOM MC were significantly ↑ below the inflammatory band, and MC degranulation was also found to be prominent
2010^16^	OSCC	MC count MVD	MC count and MCD significantly increased from normal to OSCC
2011^17^	OLP	*In vitro* to *in vivo* methods compared to evaluate angiogenetic phenomenon	Neoangiogenesis occurs in OLP
2011^18^	OLP Oral lichenoid reaction	MC count	MC count ↑ in OLP and OLR in comparison to NOM
2012^19^	Different grades of dysplasia Different grades of dysplasia OSCC	MCD	VEGF and MVD increased with disease progression and were statistically higher in oral OSCC than in epithelial dysplasia and NOM
2012^9^	OSCC	MCD MVD	Significant difference between mean MCD and MVD values in normal mucosa and OSCC, no positive correlation observed between MCD and MVD values in OSCC
2012^20^	OLP Oral lichenoid lesions	Number of MC Thickness of epithelium and basement membrane	Number of degranulated MC in reticular layer of corium in lichenoid lesions greater than that of OLP
2012^21^	OLP	MVD	Significant angiogenesis ↑ in OLP as compared to NOM, and in erosive OLP as compared to reticular OLP
2012^22^	OLP Oral lichenoid mucositis	Number of intact MC and degranulated MC Number of capillaries Number of eosinophils	Significant ↑ in number of MC in OLP and oral lichenoid mucositis compared to NOM Significant ↑ of intact MC subepithelially within inflammatory cell infiltrate in OLP compared to oral lichenoid mucositis Significant ↑ of degranulated MC, eosinophil densities, and number of capillaries in oral lichenoid mucositis compared to OLP
2012^23^	NOM Different grades of dysplasia	MCD MVD	Exponential ↑ in MVD as MCD ↑ as it progresses from NOM to dysplasia
2013^24^	OSCC Verrucous carcinoma	MCD MVD	Significant correlation in mean MVD and mean MCD in well differentiated OSCC No significant correlation in other carcinoma grades and normal tissue
2015^25^	NOM Dysplasia OSCC	MCD MVD	MCs and MVs ↑ from NDL to DL to OSCC with the exception that MVD was slightly higher in NOM group than in NDL; this difference was not statistically significant Poor correlation between MVD and MCD
2015^26^	NOM Dysplasia OSCC	MCD MVD	MCD and MVD ↑ in oral epithelial dysplasia and in OSCC compared to NOM
2016^27^	OSCC	MCD MVD	Significant correlation found between MCD and MVD
2017^28^	NOM Oral leukoplakia (without and with dysplasia) OSCC	MCD MVD	MVD and MCD significantly ↑ in OSCC cases as compared to leukoplakia with and without dysplasia and normal gingival tissue
2018^2^	OSCC	MCD MVD	Significant correlation between MCD and MVD in OSCC; both are significantly ↑ in disease process compared to that of NOM
2018^29^	NOM Dysplasia OSCC	MC count	Highly significant ↑ of MC in oral epithelial dysplasia compared to OSCC Significant ↑ in MC in OSCC compared to NOM
2018^30^	OLP OLR	MC count	MC count ↑ in OLP and OLR compared to NOM
2019^31^	OLP	MVD	Vascular density ↑ in OLP could be result of angiogenesis phenomenon, and mediators of angiogenesis are potential markers for lesion progrssion toward dysplastic changes

DL, dysplastic lesion; MC, mast cell; MCD, mast cell density; MV, microvessel; MVD, microvessel density; NDL, non-dysplastic lesion; NOM, normal oral mucosa; OLP, oral lichen planus; OLR, oral lichenoid reaction; OSCC, oral squamous cell carcinoma.

## MATERIALS AND METHODS

### Sample Collection and Distribution

Our study included a total of 75 samples, of which 65 were archival tissue blocks of histopathologically diagnosed cases of dysplasia (mild, moderate, and severe) and OSCC (well differentiated, moderately differentiated, and poorly differentiated) from the Department of Oral Pathology & Microbiology, KSR Institute of Dental Science and Research, Thiruchengode, India and AB Shetty Memorial Institute of Dental Science and Research, Mangalore, India.

Ten tissue samples from normal oral mucosa were obtained from lesion-free margins without any signs of inflammation, during oral surgeries in KSR Institute of Dental Science and Research, Thiruchengode. Samples with insufficient amounts of tissue were excluded from the study.

The present study protocol was analyzed and approved by the institutional ethical committee and review board where the study was conducted (KSR Institute of Dental Science and Research, Thiruchengode). Sample distribution is shown in Table 2.

**Table 2:** Distribution of Samples.

Lesion	Number of Samples
Normal Oral Tissues	10

Oral Lichen Planus	10

Dysplasia	25
Mild	10
Moderate	10
Severe	5

OSCC	30
WDSCC	10
MDSCC	10
PDSCC	10

MDSCC, moderately differentiated squamous cell carcinoma; OSCC, oral squamous cell carcinoma; PDSCC, poorly differentiated squamous cell carcinoma; WDSCC, well differentiated squamous cell carcinoma.

### Preparation of the Slides and Immunohistochemistry

Tissue sections of 3 to 4 μm thickness were made from the archival tissue blocks using a semiautomatic microtome. The sections were placed on APES (amino-propyl-tri-ethoxy-silane) pre-coated slides.

All the sections were immunohistochemically stained using mouse monoclonal anti-CD34 antibody (QBend/10) (ready to use, BioGenex, Fremont, CA, USA) and Super Sensitive Polymer-HRP IHC Detection System HRP/DAB (BioGenex, Fremont, CA, USA) to detect the blood vessels.

### Counterstaining with Freshly Prepared Toluidine Blue

Stock solution toluidine blue was prepared by adding 1 g of toluidine blue to 100 mL of 70% ethanol. Then, a sodium chloride solution was prepared by dissolving 0.5 g of sodium chloride in 50 mL of distilled water to make 1% sodium chloride. The solution has to be made fresh each time before staining. Then, a working solution of toluidine blue was prepared by adding 5 mL of stock solution toluidine blue to 45 mL of 1% sodium chloride. The solution was made fresh and discarded after each use.

All the immunohistochemically stained slides were counterstained with freshly prepared toluidine blue (working solution) to stain the mast cells, and then washed thoroughly with distilled water to remove the excess.

The sections were dehydrated in 50% alcohol, followed by clearing in xylene and covered by coverslip using media dibutyl phthalate xylene (DPX), a non-aqueous permanent mounting medium.

We preferred using anti-CD34 because it is a pan-endothelial marker which will stain intensely with the lowest background staining.^21^ Counterstaining with freshly prepared toluidine blue helps us to detect and count the mast cells and microvessels in the same optical field. This method has been used by only few studies in the literature.^14^,^25^ Thus this method aided us in determining the mast cells and microvessels together and helped us to determine their association.

### Evaluation of Slides

All the stained sections were observed using a binocular light microscope (CH20i, Olympus, made in Noida, India under license from Olympus Corporation, Tokyo, Japan) at 10× magnification, and, with a one-field depth from the basement membrane of the epithelium or from the tumor invasive margins or from the tumor nests, four areas with the highest number of blood vessels (hotspots) were selected. The microvessel density (number of microvessels per optical field) was determined by counting the number of blood vessels in each field at 40× magni-fication, and the average was recorded. During counting, single endothelial cells, clusters of endothelial cells, and endothelial cells lining the lumen which turned a brown or black color with anti-CD34 were considered positively stained blood vessels. Simultaneously, mast cell density (the number of mast cells per optical field) was determined by counting the mast cells in the same four already selected microscopic fields, at 40× magnification, and the average was recorded for each sample. Mast cell granules, appearing violet with bluish background and clearly separated from adjacent clusters, were considered as single mast cells and were counted in each field; the average of the four fields was recorded. All the evaluations were performed by a single investigator.

### Statistical Analysis

Mean mast cell density (MCD) and microvessel density (MVD) were determined for each lesion, and the values were statistically analyzed using ANOVA test and compared between the lesions using Tukey HSD test. Correlation of these two factors was done using Pearson’s correlation coefficient test.

## RESULTS AND OBSERVATIONS

This study was comprised of 65 archival tissue blocks of diagnosed cases of dysplasia (mild, moderate, and severe) and OSCC (well differentiated, moderately differentiated, and poorly differentiated), and 10 tissue samples of normal oral mucosa.

The mean MCD and the mean MVD in different lesions and intergroup comparison of MCD and MVD are shown in Table 3 and Table 4.

**Table 3:** Mean Mast Cell Density (MCD) and Mean Microvessel Density (MVD) in Different Lesions.

Lesion	Mean MCD	SD	*P* Value	Mean MVD	SD	*P* Value
Normal Oral Tissues	2.8250	1.71614	0.000*	9.3500	1.79969	0.000*
	
Oral Lichen Planus	10.1500	1.77639		11.3250	2.43256	
	
Dysplasia	9.1000	3.92906		12.3700	4.21006	
Mild	6.5750	2.27013		10.1000	3.63280	
Moderate	10.1500	4.73932		12.9000	4.50802	
Severe	12.0500	0.95851		15.8500	1.48535	
	
OSCC	14.5083	3.81551		15.1000	3.57771	
WDSCC	13.600	5.21243		13.8500	3.69722	
MDSCC	13.7750	2.16169		14.2000	3.10421	
PDSCC	16.1500	3.24936		17.2500	3.18852	

* *P*<0.05 significant; ANOVA test.

MDSCC, moderately differentiated squamous cell carcinoma; OSCC, oral squamous cell carcinoma; PDSCC, poorly differentiated squamous cell carcinoma; WDSCC, well differentiated squamous cell carcinoma.

**Table 4:** Intergroup Comparison of Mean Mast Cell Density (MCD) and Mean Microvessel Density (MVD) Between the Lesions.

Lesion	MCD *P* Value	MVD *P* Value
Normal Oral Tissues vs Oral Lichen Planus	0.000*	0.594
Normal Oral Tissues vs Dysplasia	0.000*	0.109
Normal Oral Tissues vs OSCC	0.000*	0.000*
Lichen Planus vs Dysplasia	0.849	0.857
Oral Lichen Planus vs OSCC	0.005*	0.023*
Dysplasia vs OSCC	0.000*	0.027*

* *P* value at <0.05 significant; Tukey HSD test.

OSCC, oral squamous cell carcinoma.

In this study, the mean MCD in normal oral tissues, oral lichen planus, dysplasia, and OSCC were 2.8250, 10.1500, 9.1000, and 14.5083, respectively (Table 3). It was found to be increased significantly (*P*<0.000) in OSCC compared to normal tissues, and also within all grades of dysplasia (mild, moderate, and severe) and also within all grades of OSCC (well, moderately, and poorly differentiated). The MCD in lichen planus was found to be higher than in dysplasia. While comparing MCD between the lesions, the Tukey HSD test revealed significant differences between all the lesions except oral lichen planus and dysplasia (*P*=0.849) (Table 4).

Results showed that the mean MVD in normal oral tissues, oral lichen planus, dysplasia, and OSCC were 9.3500, 11.3250, 12.3700, and 15.1000, respectively. It was found to be increased significantly (P<0.000) in OSCC compared to normal tissue and also within all grades of dysplasia and OSCC (Table 3).

ANOVA test on the MVD values of all lesions revealed statistically significant differences (*P*=0.000) (Table 3), and when comparing MVD within the lesions Tukey HSD test revealed significant differences only between normal oral mucosa and OSCC (*P*=0.000) and oral lichen planus and OSCC (*P*=0.023), whereas we found no significant differences between other lesions (Table 4).

**Figure 1:**
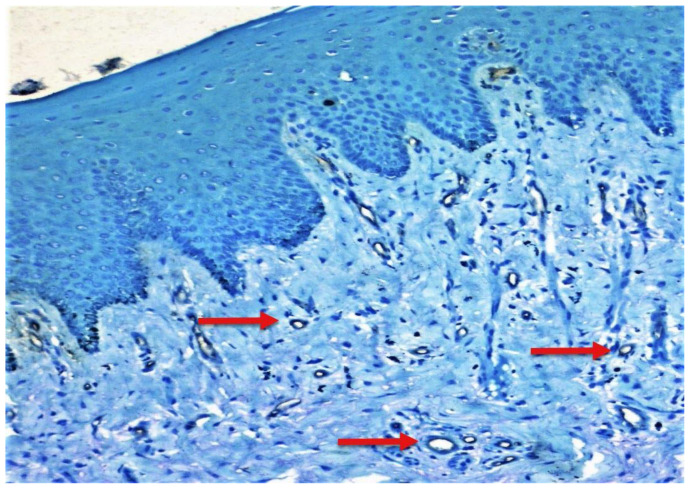
Normal Oral Mucosa Photomicrograph (10×) showing CD34-positive blood vessels (red arrows) in normal oral mucosa.

**Figure 2:**
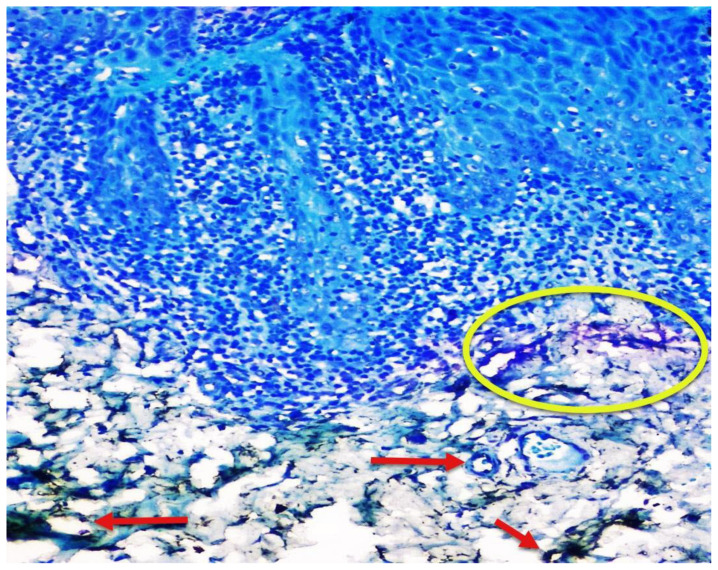
Oral Lichen Planus Photomicrograph (10×) showing CD34-positive blood vessels (red arrows) and toluidine blue-stained mast cells (yellow circle) in oral lichen planus.

**Figure 3:**
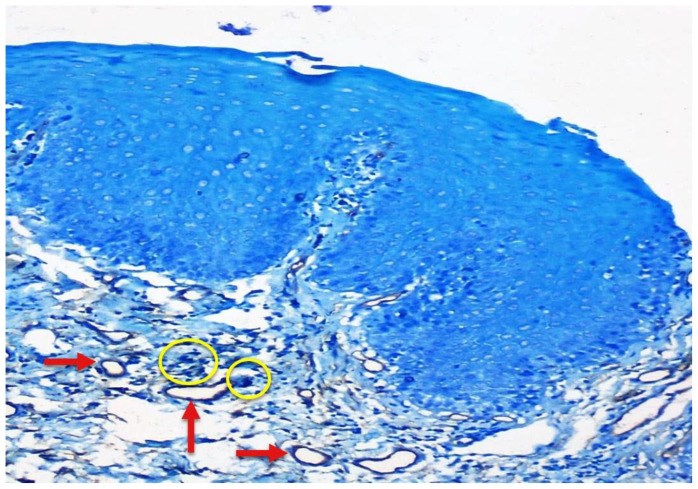
Severe Dysplasia Photomicrograph (10×) showing CD34-positive blood vessels and toluidine blue-stained mast cells in severe dysplasia.

**Figure 4:**
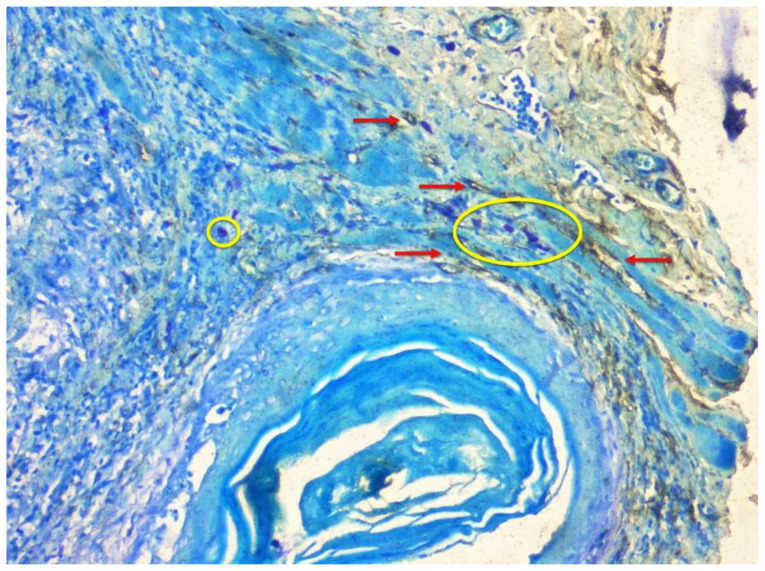
Well Differentiated Squamous Cell Carcinoma Photomicrograph (10x) showing CD34-positive blood vessels (red arrows) and toluidine blue-stained mast cells (yellow circles) in well differentiated squamous cell carcinoma near keratin pearl and tumor invasive front.

**Figure 5:**
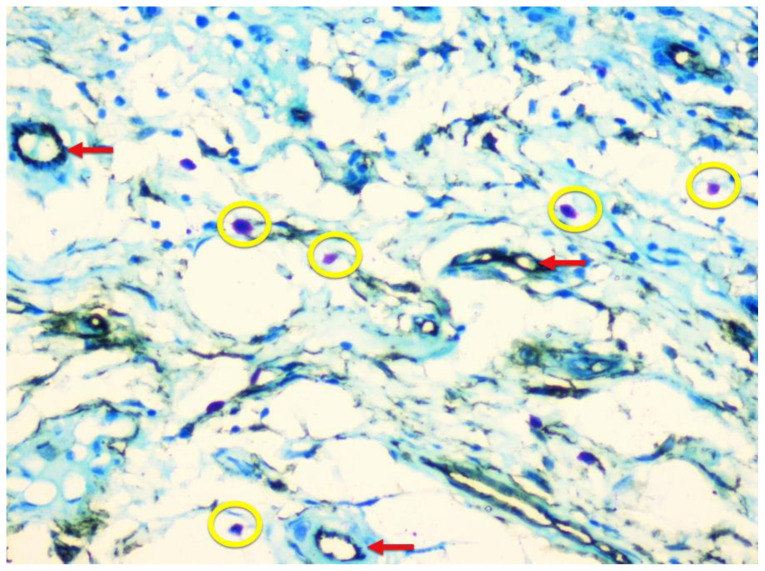
Mild Dysplasia Photomicrograph (20×) showing CD34-positive blood vessels (red arrows) and toluidine blue-stained mast cells (yellow circles) in mild dysplasia.

**Figure 6:**
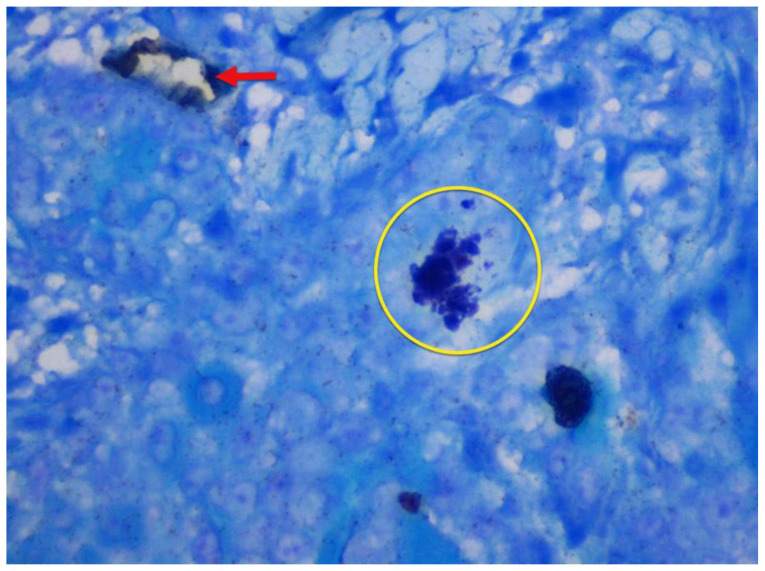
Well Differentiated Squamous Cell Carcinoma Photomicrograph (40×) showing CD34-positive blood vessels (red arrow) and toluidine blue-stained degranulated mast cells (yellow circle) in well differentiated squamous cell carcinoma.

Few mast cells were found in normal mucosa; they were found near the basement membrane, adjacent to the blood vessels (Figure 1). In lichen planus, the numerous mast cells were also found to accumulate close to the basement membrane below the inflammatory infiltrate and near the blood vessels (Figure 2). In dysplasia, numerous mast cells were found close to the basement membrane and near the blood vessels (Figure 3). In OSCC, mast cells were found close to the tumor islands and keratin pearls, and they accumulated near the blood vessels (Figure 4). Numerous granulated (Figure 5) and degranulated mast cells were found (Figures 6).

Correlation between MCD and MVD in each lesion was analyzed using Pearson’s correlation coefficient test (Table 5), and this showed a strong positive correlation (*r*=0.767) and a highly statistically significant (*P*=0.0001) difference in dysplasia. Mild positive correlation was seen in oral lichen planus (*r*=0.103) and OSCC (*r*=0.270), but the values were not statistically significant. No correlation was found in normal mucosa, and the results were not statistically significant (Table 5).

**Table 5:** Overall Correlations Between Mean Mast Cell Density (MCD) and Mean Microvessel Density (MVD) in Each Lesion.

Lesion	Pearson’s Correlation Value (*r*)	*P* Value	Inference
Normal Oral Tissues	−0.025 or 0	0.0945	No correlation Not statistically significant
Oral Lichen Planus	0.103	0.777	Mild positive correlation Not statistically significant
Dysplasia	0.767	0.0001*	Strong positive correlation Highly statistically significant
OSCC	0.270	0.149	Mild positive correlation Not statistically significant

* *P*<0.05 significant.

OSCC, oral squamous cell carcinoma.

**Figure 7:**
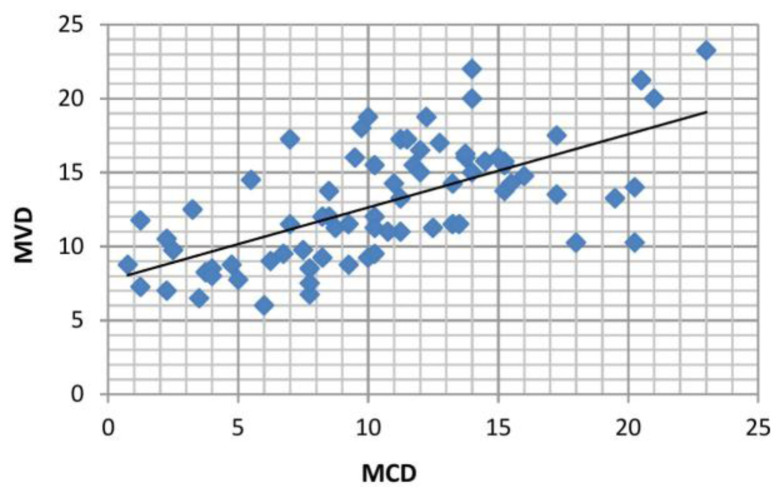
Overall Correlation between Mean Mast Cell Density (MCD) and Mean Microvessel Density (MVD).

Overall correlation between MCD and MVD was analyzed using Pearson’s correlation coefficient, and this showed a positive correlation (*r*=0.640), and *P* value was found to be highly significant (*P*=0.0001), as shown in Table 6 and Figure 7.

**Table 6:** Overall Correlation of Mean Mast Cell Density (MCD) and Mean Microvessel Density (MVD).

Correlation	Value
*r* Value	0.640
*P* Value	0.0001*

* *P*<0.05 significant.

*r* value, Pearson’s correlation value.

## DISCUSSION

Tumor growth and metastasis are angiogenesis-dependent. The process of angiogenesis depends on many angiogenic factors produced by the tumor and the tumor microenvironment.^14^ The tumor microenvironment is a mixture of extracellular matrix molecules, tumor cells, endothelial cells, fibroblasts, and immune cells and plays an important role in tumorigenesis. Mast cells are one type of inflammatory cells which produce a large spectrum of pro-angiogenic factors and promote angiogenesis.^10^

In 1878, Paul Ehrlich described mast cells as metachromatic, granulated cells implicated in the nutrition of the surrounding tissue and named them “Mastzellen.” In 1937, Holmgren and Willanderm first observed that tissues that displayed a great number of “Ehrlichschen Mastzellen” (mast cells) were enriched in heparin.^32^ These cells are found resident in tissues throughout the body, particularly in association with blood vessels and nerves, and in the surfaces that are in close contact with the external environment.^33^ Mast cells (MCs) accumulate in many angiogenesis-dependent situations both in physiological and pathological situations.^10^ Degranulation of mast cells releases proinflammatory mediators such as tryptase, chymase, heparin, histamine, TNF-α, MMPs, basic fibroblast growth factor (bFGF), various interleukins (IL-3, IL-4, IL-5, IL-6, IL-8, IL-10, IL-13, and IL-16), cytokines, and RANTES; it also produces a large spectrum of pro-angiogenic factors which promotes angiogenesis.^10^ Degranulation is induced by various stimuli like IgE receptors, chemokines, neuropeptides, and other physical stimuli. Once these compounds are released into the extracellular environment they can have a marked effect on any physiological or pathophysiological event.^34^

Several studies have found correlations between angiogenesis and tumor progression in different oral lesions and suggested that host immune cells, particularly mast cells, have a role as they promote angiogenesis (Table 1).

In our present study, we found that the mean MCD was significantly increased in oral lichen planus, dysplasia, and OSCC compared to normal mucosa. It was also increased in all grades of dysplasia and OSCC. This result was similar to that of previous studies.^14^,^19^,^23^,^26^,^28^,^35^

According to Janardhanan and Ramesh, on degranulation mast cells release a range of pro-inflammatory mediators such as histamines, TNF-α, chymase, and tryptase, and each of these mediators has specific functions in the pathogenesis of oral lichen planus.^15^ Histamine causes dilatation of the blood vessel and extravasation of the lymphocytes during the initial phase. It also releases some cytokines that cause destruction of the extracellular matrix and attract the targeting lymphocytes toward the basement membrane.^15^

In our present study, MCD was found to be higher in lichen planus than in dysplasia. This finding was in accordance with Ankle et al.^13^ and Kinra et al.^36^ In dysplasia, the stimulated mast cells releases interleukin-1, causing increased epithelial proliferation which may help in disease progression.^36^

The results of our present study showed that mast cell count was increased in squamous cell carcinoma compared to lichen planus. Heparin from the mast cells cause vasoproliferation and increase the half-life of basic fibroblastic growth factor (FGF), which is a potent angiogenic substance, thereby promoting tumor angiogenesis and facilitating local tumor invasion. Interleukin-1 released by mast cells also leads to epithelial proliferation.^36^

Results from the present study showed that the mean MVD was significantly increased in lichen planus, dysplasia, and OSCC compared to normal mucosa. Our results also showed significantly increased mean MVD within all grades of dysplasia and OSCC. These results were in accordance with previous studies.^14^,^19^,^23^,^26^,^28^,^35^

The MVD was found to be increased in oral lichen planus compared to normal oral mucosa, which was consistent with previous studies conducted by Scardina et al.^17^ and Mittal et al.^21^ According to Mittal et al., proangiogenic and angiogenic factors such as histamine, heparin, chymase, bFGF, VEGF, and TGF-beta that are produced by mast cells increase angiogenesis, which in turn will lead to more recruitment and retention of lymphocytes or inflammatory infiltrate, leading to the progression of disease or recurrence of the lesions.^17^,^21^ Intergroup comparison was carried out between oral lichen planus and dysplasia. The increase of MVD in dysplasia might suggest that angiogenesis has a role in disease progression but this needs further research with larger samples. Intergroup comparison of MVD in oral lichen planus and OSCC was carried out, and we found that MVD was significantly increased in OSCC compared to oral lichen planus. This finding in our study might suggest that angiogenesis has a role in disease progression, but this needs further research.

We observed an increased MVD in dysplasia compared to normal oral mucosa; however, the increase in MVD was not statistically significant, which is consistent with Khare et al.^25^ Among dysplasias, MVD was found to be higher in severe dysplasia compared to moderate and mild dysplasia, and this finding was consistent with the study conducted by Michailidou et al.,^14^ Astekar et al.,^19^ and Sathyakumar et al.^23^ This supports the concept that the increased nutrient requirement of actively growing and dividing cells increases angiogenesis, which in turn plays a role in tumor progression.^14^,^23^

Intergroup comparison of MVD in dysplasia and OSCC was carried out. It was found that the increase in MVD was statistically significant and consistent with studies conducted by Michailidou et al.,^14^ Mohtasham et al.,^35^ Astekar et al.,^19^ Hegde et al.,^26^ and Khare et al.^25^

Among OSCC, MVD was found to be higher in poorly differentiated OSCC compared to moderately differentiated OSCC and well differentiated OSCC, which was consistent with studies done by Devi et al.^24^ and Ingaleshwar et al.^27^ Thus, the increased MVD in OSCC compared to normal oral mucosa and also between all grades of OSCC showed the role of angiogenesis during the tumor progression.

The present study showed a positive Pearson’s correlation between MCD and MVD in normal oral mucosa, potentially malignant disorders, and squamous cell carcinoma. There was a weak/positive correlation between MCD and MVD in oral lichen planus, which was found to be not statically significant. Based on our results, the role of mast cells in promoting angiogenesis is less central, or it may be that other factors are involved in the pathogenesis of lichen planus—a topic that needs further research. Our results showed a strong positive correlation between MCD and MVD in dysplasia. This was found to be highly statistically significant and consistent with the results by Sathyakumar^23^ and Hegde et al.^26^ In OSCC, our results showed a mild positive correlation between MCD and MVD that was not statistically significant, consistent with the results of Hegde et al.^26^ and Nakandala et al.^11^ The mild positive correlation may be due to the failure of migration of mast cells, or factors other than mast cells probably involved in tumor angiogenesis.^11^ In other words, initially, mast cells might infiltrate cancerous tissue to suppress tumor activities, but, once invasion occurs, their cytotoxic effects are nullified, thereby enhancing tumor progression rather than supporting angiogenesis.^26^ This mast cell-mediated cytotoxicity was reported when the mast cell:tumor ratios are greater than 20:1. Thus, the effect of mast cells on cancer cells might depend on the concentration of mast cell products in the microenvironment.^37^ Thus, mast cells have a dual role of promoting angiogenesis and invasion in tumor progression. Therefore, reversing this effect, i.e. enhancing the cytotoxic functions of mast cells and suppressing their angiogenic functions, could lead to a new anti-cancer treatment strategy.^37^

Overall correlation analysis between MCD and MVD was carried out in all the lesions considered for this study and showed a moderate positive correlation. Both parameters showed highly significant differences, which indicates their role in tumor progression.

## CONCLUSION

From our findings and previous literature, there is sufficient evidence to suggest the role of mast cells in tumor pathogenesis, angiogenesis, and progression. But further studies with larger samples and with better methods for identification of mast cells and blood vessels are still needed for a better understanding of the exact role of mast cells in oral lichen planus and in tumor progression.

## Abbreviations

MCD: mast cell density
MVD: microvessel density
OSCC: oral squamous cell carcinoma.
